# Designing of inhibitors against drug tolerant *Mycobacterium tuberculosis* (H37Rv)

**DOI:** 10.1186/1752-153X-7-49

**Published:** 2013-03-08

**Authors:** Deepak Singla, Rupinder Tewari, Ashwani Kumar, Gajendra PS Raghava

**Affiliations:** 1 Bioinformatics Centre, CSIR-Institute of Microbial Technology, Sector 39A, Chandigarh, India; 2 Infectious Diseases and Immunology, CSIR-Institute of Microbial Technology, Chandigarh, India; 3 Centre For Microbial Biotechnology, Panjab University, Chandigarh, India; 4 CSIR Open Source Drug Discovery Unit, Anusandhan Bhavan, 2 Rafi Marg, Delhi, 110001, India

## Abstract

**Background:**

*Mycobacterium tuberculosis* (*M.tb*) is the causative agent of tuberculosis, killing ~1.7 million people annually. The remarkable capacity of this pathogen to escape the host immune system for decades and then to cause active tuberculosis disease, makes *M.tb* a successful pathogen. Currently available anti-mycobacterial therapy has poor compliance due to requirement of prolonged treatment resulting in accelerated emergence of drug resistant strains. Hence, there is an urgent need to identify new chemical entities with novel mechanism of action and potent activity against the drug resistant strains.

**Results:**

This study describes novel computational models developed for predicting inhibitors against both replicative and non-replicative phase of drug-tolerant *M.tb* under carbon starvation stage. These models were trained on highly diverse dataset of 2135 compounds using four classes of binary fingerprint namely PubChem, MACCS, EState, SubStructure. We achieved the best performance Matthews correlation coefficient (MCC) of 0.45 using the model based on MACCS fingerprints for replicative phase inhibitor dataset. In case of non-replicative phase, Hybrid model based on PubChem, MACCS, EState, SubStructure fingerprints performed better with maximum MCC value of 0.28. In this study, we have shown that molecular weight, polar surface area and rotatable bond count of inhibitors (replicating and non-replicating phase) are significantly different from non-inhibitors. The fragment analysis suggests that substructures like hetero_N_nonbasic, heterocyclic, carboxylic_ester, and hetero_N_basic_no_H are predominant in replicating phase inhibitors while hetero_O, ketone, secondary_mixed_amine are preferred in the non-replicative phase inhibitors. It was observed that nitro, alkyne, and enamine are important for the molecules inhibiting bacilli residing in both the phases. In this study, we introduced a new algorithm based on Matthews correlation coefficient called MCCA for feature selection and found that this algorithm is better or comparable to frequency based approach.

**Conclusion:**

In this study, we have developed computational models to predict phase specific inhibitors against drug resistant strains of *M.tb* grown under carbon starvation. Based on simple molecular properties, we have derived some rules, which would be useful in robust identification of tuberculosis inhibitors. Based on these observations, we have developed a webserver for predicting inhibitors against drug tolerant *M.tb* H37Rv available at http://crdd.osdd.net/oscadd/mdri/.

## Introduction

Tuberculosis (TB), a disease caused by *M.tb* kills around 1.7 million people every year despite the availability of effective chemotherapy for more than half a century [1]. The antibiotic resistant strains of *M.tb* have arisen primarily due to poor compliance resulting from prolonged therapy [2]. The emergence of multiple drug-resistant (MDR), extensive drug-resistant (XDR) strains, and its association with HIV has severely affected the fight against TB [3]. Mathematical models have predicted that the MDR-TB and XDR-TB epidemics have the potential to further expand, thus threatening the success of TB control programs attained over last few decades [4-6].

In humans, the pathogenic cycle of TB consists of three phases [7]: i) an active TB disease phase with actively replicating bacteria; ii) a latent phase wherein bacteria achieves a phenotypically distinct drug resistant state; and iii) a reactivation phase. The active TB disease phase is characterized by exponential increase of the pathogen, and latent phase is characterized by dormant phase in which pathogen remains metabolically quiescent and is not infectious. However, the reactivation phase is characterized by transition of latent infection into active TB disease. The reactivation of the disease occur in nearly 10% of patients with functional immune system and no separate dataset of inhibitors for this phase of pathogenic cycle is available. Therefore, in this study, we have used two phase inhibitors namely active and latent phase.

In past, researchers across the globe have deposited high throughput experimental data from *M.tb* growth inhibition assays. In PubChem, numerous datasets consisting of both the specific target based as well as cell-based inhibition assays are available. Utilizing these datasets, few computational models have been developed in past [8-11]. However, these studies are of little significance as they failed to contemplate the effect of potential hits on the drug-resistant *M.tb* strains grown under nutrient starvation condition. Furthermore, these studies does not distinguish the inhibitors based on their activity in different phase of TB. Therefore, it is important to develop new theoretical models for predicting inhibitors that would be effective against replicative as well as non-replicative drug-resistant *M.tb* and could potentially treat active TB patients as well as latently infected individuals.

Experimental techniques used in identification of inhibitors of *M.tb* growth are very expensive, time-consuming, tedious and requires sophisticated infrastructure (BSL-3) for mitigation of risk of infection. Thus, there is an urgent need to develop *in-silico* models for predicting inhibitors against drug-tolerant *M.tb*. In past, a number of target based models have been developed using QSAR and docking [12-16] for identification of novel inhibitors against *M.tb*. However, impermeability of chemical compounds to the mycobacterial cell wall hindered them to act as good lead molecules. To the best of our knowledge, no attempt has been made to develop prediction models against phase specific drug-tolerant *M.tb*.

Despite the enormous progress in computational and medicinal chemistry, only few webservers namely KiDoQ [17], GDoQ [18] and CDD [19] for predicting the efficacy of potential antimycobacterial drug like molecules are freely available to the scientific community. In order to assist researchers in discovering new chemical entity (NCE) against tuberculosis, a systematic algorithm has been developed to predict the inhibitors of replicative and non-replicative drug tolerant *M.tb* H37Rv*.*

## Methods

### Data source

**Figure 1:**
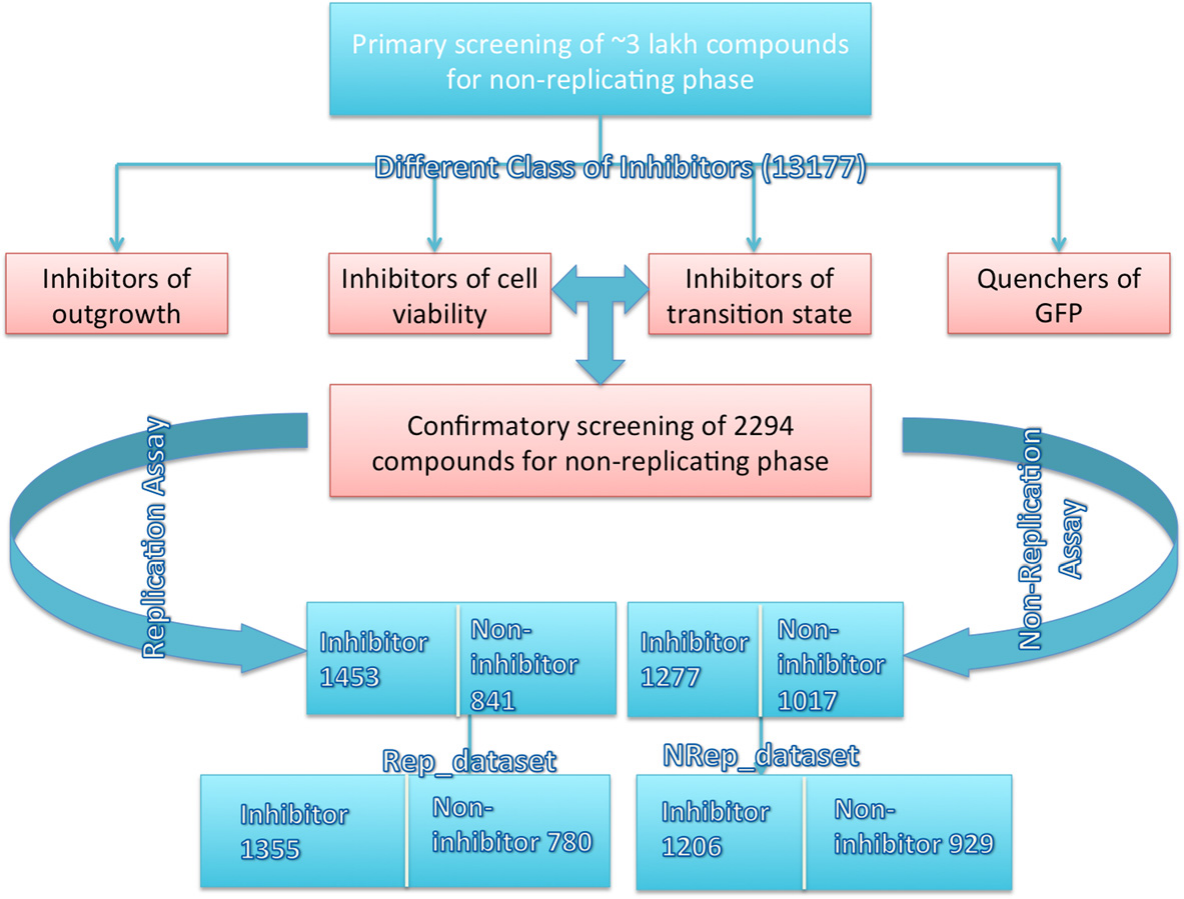
Showing the flow diagram of datasets.

Datasets were created from PubChem confirmatory BioAssay [AID-492952 (replicating), and AID-488890 (non-replicating)] screens of drug tolerant *M.tb* H37Rv in carbon starvation model [20,21]. Although in past, hypoxia induced model have been used for compound screening but only AID-488890 has been used to study carbon starvation model of persistence. Since, the behaviour of compounds is different under different physiological conditions, therefore it is extremely important to identify and explore the structure activity relationship (SAR) of inhibitors against this pathogen in carbon starvation stage. The BioAssay (AID-488890) involved primary screening of more than 3 lakh compounds that identified 13,177 active compounds. This screening identified four classes of inhibitors: 1) inhibitors of viability under carbon-starvation, 2) inhibitors of transition from carbon-starved to replicating state, 3) inhibitors of outgrowth, and 4) quenchers of GFP fluorescence used as reporter of outgrowth. From these 13177 compounds, a total of 2294 compounds were selected for confirmatory screening based on class-I and class-II inhibitors. In both BioAssay, compounds that showed >30% inhibition for at least one concentration were defined as “Active”, otherwise defined as “Inactive”. All the compounds used in these assay were downloaded in SDF format, processed and named Rep_dataset (replicating), and NRep_dataset (non-replicating) as described below in detail [Figure 1].

#### NRep_dataset

The confirmatory screening in this assay resulted in 1277 active and 1017 inactive compounds against non-replicating *M.tb*. After removing the compounds containing salt/ions, we got a final dataset of 2135 compounds, out of which 1206 were identified as inhibitors and 929 were non-inhibitors.

#### Rep_dataset

This dataset involved screening of 2294 compounds from the BioAssay-488890 and identified 1453 inhibitors and 841 non-inhibitors for *M.tb* residing in replicating phase. After removing the salt/ions containing compounds, the final dataset was composed of total 2135 compounds of which 1355 compounds acted as replication mode inhibitor and rest were non-inhibitors.

### SMART filters

The SMART pattern is the fragments present in compounds with undesirable effect reported in past and found to be responsible for toxicity or other side-effects. Therefore, it is important to search these reactive, non-advisable functional groups in the compounds with drug-like potential. In this study, we have used SMART filter web application (http://pasilla.health.unm.edu/tomcat/biocomp/smartsfilter) with Abbott ALARM [22], Glaxo [23] and Pfizer LINT [24] SMART filters. In this software, each compound was evaluated for potential to pass each particular filter. A molecule matching to this filter is classified into the failed category. On this basis, it will identify the number of compounds that pass or fail any of the implemented filters.

### Substructure fragment analysis

In order to mine the hidden structural motifs present in chemical compounds, in this study, we have used the substructure pattern recognition method as described by Shen *et.al*[25,26]. The dictionary of SubFP (substructure fingerprints) containing 307 substructure (SMART) pattern, which is freely available in PaDEL software was used. These patterns were analyzed by substructure fragment analysis [27]. The frequency of a fragment in the inhibitors and non-inhibitors of *M.tb* for a particular phase was calculated as follows.

(1) Frequencyofafragment=Nfragment_phaseXNtotalNfragment_totalXNclass

where N_fragment_phase_ is the number of compounds containing the fragment in a *M.tb* phase inhibitor. N_total_ is the total number of compounds in that phase, N_fragment_total_ is the total number of compounds containing the fragment, and N_class_ is the number of compounds in the *M.tb* phase inhibitor.

### Pharmacophore search

Since the pharmacophore represents the critical point present in chemical structure and take part in protein interaction, thus we have explored these features present in our datasets. The pharmacophore features were generated for the three first line (rifampicin, ethambutol and streptomycin) and four second line (ethionamide, cycloserine, kanamycin, amikacin) *M.tb* drugs using pharmagist software [28]. These pharmacophores (named pharmacophore-1, pharmacophore-2) were then used to search similar compounds among the inhibitors of Rep_dataset, and NRep_dataset.

### Descriptor calculations

The PaDEL software has the capability of calculating 10 different types of fingerprints and 813 2D-3D descriptors [29]. The binary fingerprints are easy to calculate, informative and interpretable, therefore we have used these in our datasets (see Data source section). The bit-string fingerprint is represented by 0’s or 1’s for the absence or presence of a particular fragment. In this study, we have used four different types of fingerprints.

### Descriptor selection

It has been previously recognized that amongst the huge number of descriptors, only a few are relevant for efficient model building [30]. It is well known that the computation time increases diagonally with addition of parameters. Furthermore, some descriptors that increases the noise level tremendously affect the model quality. Therefore, selection of highly relevant descriptors is a crucial step to reduce the noise level and to build a robust classification model. Therefore, we adopted multilayer techniques by 1) removing highly correlated descriptors (> = 0.8 to > =0.4), 2) MCC based selection of descriptors, 3) frequency based selection. For example, initially calculated 881 PubChem fingerprints calculating using PaDEL software were reduced to 597 after removing useless fingerprints, then to 247 by removing highly correlated descriptors at correlation cutoff 0.6.

### Classification models

#### SVM based classification models

We have used support vector machine (SVM) for discrimination between inhibitors and non-inhibitors of drug tolerant *M.tb* for both replicative and non-replicating phases. SVM can handle complex structural features based on the statistical and optimizations theory. In optimization process, the most important parameter is kernel function and is represented by t that varies from 0, 1, 2 corresponds to linear, polynomial, and radial basis function (RBF). The purpose of kernel function is to build a hyperplane that could separate two classes of data more accurately. For RBF kernel, the other parameter values are g, c, and j where c is used to trade-off between training error and margin, j is used to assign the cost, important in imbalance dataset and g is the gamma factor. In this study, we used SVM^light^ software package, which is freely available and can be downloaded from http://www.cs.cornell.edu/People/tj/svm_light/. The performance of models was optimized using a systematic variation of these different SVM parameters and kernels.

### Evaluation of performance

To evaluate the performance of the prediction model, we adopted a five-fold cross validation approach. In this approach, the whole data was divided into five sets. Four sets were used in training and remaining 5th set was used for testing. This process was repeated five times such that each set comes in test set one time. If a particular compound was active and the prediction also envisage the same, then this was classified as true positive (TP); if actual was active and prediction was inactive, then it was false negative (FN); if actual was inactive and prediction was active, then its false positive (FP); and if actual is inactive and prediction is also inactive, then it’s true negative (TN) [26]. Once the model was constructed fitness of the model was assessed using the commonly used statistical parameters [26]. We have also created receiver operating curve (ROC) to evaluate the performance of models using threshold independent parameters. ROC plots with area under the curve were created using ROCR package in R.

## Results

This study is based on high-throughput screening data from PubChem BioAssay for identifying potential inhibitors against drug tolerant *M.tb* H37Rv (replicative phase and non-replicative phase).

### Analysis of inhibitors and non-inhibitors

We calculated the descriptors of both Rep_dataset and NRep_dataset (see Methods section) using the Marvin plugin (ChemAxon, Budapest, Hungary (http://www.chemaxon.com). We observed that the mean value of molecular weight, Atom count and number of rotatable bonds (RBN) was significantly higher (*p* < 0.05) in inhibitors of replicating phase as compared to non-inhibitors whereas the lower mean value of these descriptors was observed in case of inhibitors of non-replicating phase as compared to non-inhibitors [Table 1]. We compared inhibitors of both replicating and non-replicating phase, and observed that molecular weight, hydrogen bond acceptor, atom count, polar surface area, and rotatable bond count is significantly lower (*p* < 0.05) in the compounds inhibiting replication phase of *M.tb.* Furthermore, we analyzed these important properties to identify any correlation between these descriptors and activity and derived new rules for identifying inhibitors of mycobacterial growth [see detail in Additional file 1]. Our analysis suggested that the percentage of inhibitors against mycobacterial growth (replicative phase) were more as compared to percentage of non-inhibitors when the molecular weight is >300 Da. This means probability of being inhibitors in this range is higher as compared to non-inhibitors (see Additional file 1: Figure S1). Likewise, the percentage of active compounds were more in comparison to percentage of inactive when the hydrogen bond acceptor is >5 and rotatable bond count >6 (see Additional file 1: Figure S3, Additional file 1: Figure S5). Similarly, when polar surface area was <88 Å, percentage of decoys were more as compared to percentage of active molecules implies that the compounds with polar surface area >88 Å were preferred for inhibitors [see Additional file 1: Figure S4]. However, for designing inhibitors against non-replicative mycobacteria, percentage of active is more as compared to percentage of inactive when the molecular wt. of compounds is <380 Da (see Additional file 1: Figure S6). This means that molecules with molecular weight less than 380 Da were preferred in inhibitors as compared to non-inhibitors. Similarly percentage of inhibitors was less as compared to percentage of non-inhibitors when rotatable bond was <2 and >4 (see Additional file 1: Figure S8).

**Table 1:** **Mean (SD) of molecular descriptors from the *M.tb *datasets, compared actives and inactives**

**Descriptor**	**Rep_dataset**		**NRep_dataset**		**Rep_dataset vs. NRep_dataset**	
	**Inh**^**a**^	**NI**^**b**^	**Inh**^**a**^	**NI**^**b**^	**Inh**^**a**^	**Inh**^**a**^
**Molecular weight**	325.73 (55.07)^#^	312.37 (57.71)^#^	317.32 (53.42)^#^	325.44 (59.78)^#^	317.32 (53.42)^#^	325.73 (55.07)^#^
**logP**	2.96 (0.95)^#^	2.82 (0.99)^#^	2.92 (0.93)	2.90 (1.02)	2.92 (0.93)	2.96 (0.95)
HBA^*^	3.93 (1.35)^#^	3.39 (1.29)^#^	3.76 (1.36)	3.70 (1.34)	3.76 (1.36)^#^	3.93 (1.35)^#^
HBD^**^	0.97 (0.76)	1.00 (0.76)	1.00 (0.76)	0.97 (0.77)	1.00 (0.76)	0.97 (0.76)
**Atom count**	38.31 (8.38)	37.60 (8.30)	37.19 (7.62)^#^	39.16 (9.11)^#^	37.19 (7.62)^#^	38.31 (8.38)^#^
**PSA**^!^	74.81 (27.63)^#^	64.46 (23.32)^#^	72.29 (27.17)^#^	69.38 (25.77)^#^	72.29 (27.17)^#^	74.81 (27.63)^#^
**RBN**^!!^	4.58 (2.04)^#^	4.40 (1.95)^#^	4.35 (1.91)^#^	4.73 (2.12)^#^	4.35 (1.91)^#^	4.58 (2.04)^#^

^a^*Inh*: correspond to inhibitors, ^b^*NI*: correspond to non-inhibitors, ^*^HBA: hydrogen bond acceptor, ^**^HBD: hydrogen bond donor, ^!^*PSA*: polar surface area, ^!!^*RBN*: denotes rotatable bond number, ^#^p < 0.05.

Based on these rules, we also tried to understand the behaviour of new class of anti-tuberculosis molecules and found that out of 7 replication mode inhibitors (PA-824, OPC-67683, TMC207, SQ109, Thioridazine, Lineziod, PNU-100480), on an average 3 (42.8%) molecules satisfied these rules. Similarly out of 4 inhibitors (PA-824, Thioridazine, Linezolid, Motifloxin), an average 2 (50%) compounds followed these rules. In order to further support these rules, we also analyzed 81 inhibitors (out of 177 because rest were complex form) of tuberculosis studied by Ballell *et. al.,*[31] and observed that 77.77% compounds fulfill the condition of molecular weight, 56.79% followed logP criteria, and 27.16% agreed with condition of rotatable bond count. There were only 19.75% active compounds which does not satisfy any of these rules while rest 80.25% were following one or more rules [Additional file 2: Table S1].

### Validation of dataset

**Figure 2:**
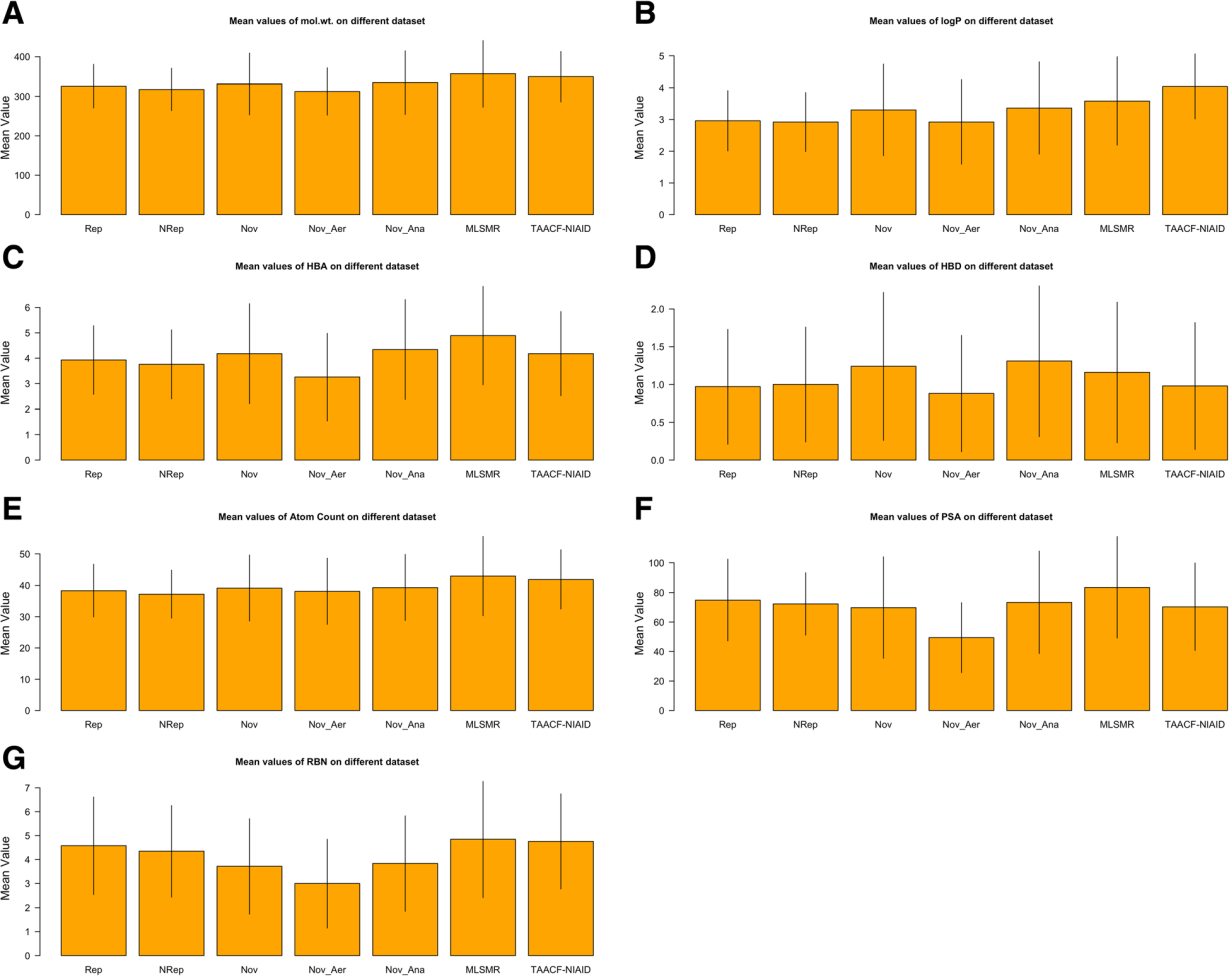
Mean molecular descriptor property values depicted in the form of column and standard deviation (SD) in the form of error bar for Rep (Rep_dataset), and NRep (NRep_dataset) inhibitors compared with Nov (Novartis), Nov_Aer (Novartis Anaerobic), Nov_Ana (Novartis Anaerobic), MLSMR and TAACF-NIAID CB2 dataset hits.

In 2011, *Ekins et. al.* used different datasets such as Novartis, MLSMR, TAACF-NIAID CB2 in their study [9]. The Novartis dataset is composed of total 283 compounds out of which 42 were aerobic and 241 were anaerobic inhibitors of *M.tb* The MLSMR and TAACF-NIAID CB2 dataset consist of 4096 and 1702 compounds responsible for inhibiting *M.tb* more than 90% at 10 μm concentration. We were interested to know the similarity of our dataset with *Ekins et.al* datasets [9]. Therefore, we computed simple molecular properties and compared in terms of the mean value and standard deviation (SD) of the descriptor [Figure 2]. The mean value of molecular weight, logP, hydrogen bond acceptor, hydrogen bond donor, and atom count is more closely related to Novartis aerobic dataset [Figure 2A-2E]. However, the polar surface area, and rotatable bond count was near to Novartis anaerobic and MLSMR dataset [Figure 2F-2G]. This means that our datasets have similar properties and are not very different from previous datasets.

### SMART filtering of the datasets

Previously, six different database namely US Antibiotic drugs from Microsource database consist of 163 compounds, US FDA drugs from Microsource contains 1041 drugs, FDA drugs from Jons Hopkins comprises of 2693 drugs, Natural Product from Microsource consist of 800 compounds, Novartis dataset consist of 283 compounds, and 13 TB drugs were used for SMART based filtering [8,9,19]. We also examined our datasets for the presence or absence of different types of filters, which were used in these datasets. It was observed that 78.6%, 16.3% and 44.1% compounds failed the Abbott ALARM, GSK, and Pfizer LINT filter respectively [Table 2]. The SMART filtering of our dataset is consistent with other datasets such as TB drugs, Novartis US antimicrobial drugs etc. [Table 2]. As observed from the Table 2, the Abbott ALARM filter has high rate of failure as compared to GSK and Pfizer LINT filters in all the different datasets.

**Table 2:** SMART filtering number of failures (%) using SMART filter website

**Filters**	**Rep_dataset**	**NRep_dataset**	**Novartis (283)**	**TB drugs (13)**	US Antibiotic^*^	US FDA^**^	**JH FDA**^**#**^	**Natural Product**^**##**^
**GSK (%)**	197 (14.5)	196 (16.3)	20 (7.1)	1 (7.7)	57 (35)	143 (13.7)	401 (14.9)	125 (15.6)
**Pfizer LINT (%)**	609 (44.9)	532 (44.1)	135 (47.7)	6 (46.1)	93 (57.0)	516 (49.6)	1264 (46.9)	304 (38.0)
**Abbott ALARM (%)**	1064 (78.5)	948 (78.6)	243 (85.9)	7 (53.8)	144 (88.3)	688 (66.1)	1442 (53.5)	521 (65.1)

^*^US Antibiotic drugs from Microsource, ^**^Microsource US FDA drugs, ^#^Jons Hopkins –All FDA drugs, ^##^Natural Product from Microsource.

### Substructure fragment analysis

To further explore the structural features responsible for killing the *M.tb*, substructure fragment analysis [25-27] was performed on both (Rep_dataset anf NRep_dataset) datasets using Substructure fingerprint (SubFP). The representative fragments characterizing the inhibitors and non-inhibitors are shown in Table 3. As shown in Table 3, pattern of hetero_O, ketone, secondary_mixed_amine, vinylogous_halide, and vinylogous_carbonyl or carboxyl_derivatives present in higher frequency in NRep_dataset inhibitors as compared to non-inhibitors, whereas no significant difference is present in case of Rep_dataset. Similarly, pattern of hetero_N_nonbasic, heterocyclic, carboxylic_ester, hetero_N basic_no_H occur more frequently in Rep_dataset inhibitors while these substructures are more or less similar in case of NRep_dataset.

**Table 3:** Frequency of 20 representative substructure fragments in the Rep_dataset and NRep_dataset

**Fragment number**	**Fragment/substructure name**	**Rep_dataset**		**NRep_dataset**	
		**F**_**I**_^#^	**F**_**nonI**_^##^	**F**_**I**_^#^	**F**_**nonI**_^##^
**SubFP181**	Hetero_N_nonbasic	**1.15**	**0.74**	1.03	0.96
**SubFP275**	Heterocyclic	**1.03**	**0.94**	1.00	1.00
**SubFP85**	Carboxylic_ester	**1.14**	**0.76**	0.97	1.03
**SubFP180**	Hetero_N_basic_no_H	**1.18**	**0.68**	0.95	1.07
**SubFP182**	Hetero_O	1.04	0.92	**1.10**	**0.87**
**SubFP49**	Ketone	0.97	1.06	**1.13**	**0.83**
**SubFP32**	Secondary_mixed_amine	1.00	1.01	**1.28**	**0.64**
**SubFP135**	Vinylogous_carbonyl or carboxyl_derivative	1.02	0.97	**1.09**	**0.88**
**SubFP139**	Vinylogous_halide	1.00	1.00	**1.16**	**0.79**
**SubFP214**	Sulfonic_derivative	0.95	1.09	0.68	1.41
**SubFP143**	Carbonic_acid_derivatives	0.95	1.08	0.80	1.27
**SubFP65**	NOS_methylen_ester_and_similar	0.41	2.03	1.38	0.51
**SubFP23**	Amine	0.92	1.14	0.70	1.39
**SubFP3**	Tertiary_carbon	0.80	1.35	0.90	1.13
**SubFP20**	Alkylarylthioether	0.69	1.55	0.79	1.27
**SubFP103**	Alkyl_imide	0.30	2.22	0.43	1.74
**SubFP2**	Secondary_carbon	0.88	1.21	0.93	1.09
**SubFP188**	Nitro	**1.23**	**0.60**	**1.14**	**0.82**
**SubFP6**	Alkyne	**1.08**	**0.86**	**1.66**	**0.14**
**SubFP76**	Enamine	**1.13**	**0.78**	**1.39**	**0.49**

^#^*F*_*I*_: Frequency of a fragment in inhibitor, ^##^*F*_*nonI*_: Frequency of fragment in non-inhibitor, ^*^bold values shows the significance of substructure in the dataset.

As shown in Table 3, the substructure patterns like nitro, alkyne, enamine were presented more frequently in case of inhibitors of both the Rep_dataset and NRep_dataset as compared to non-inhibitors. However, the patterns like amine, tertiary_carbon, alkylarylthioether and secondary_carbon are not preferred in any class of the inhibitors.

### Pharmacophore searching

**Figure 3:**
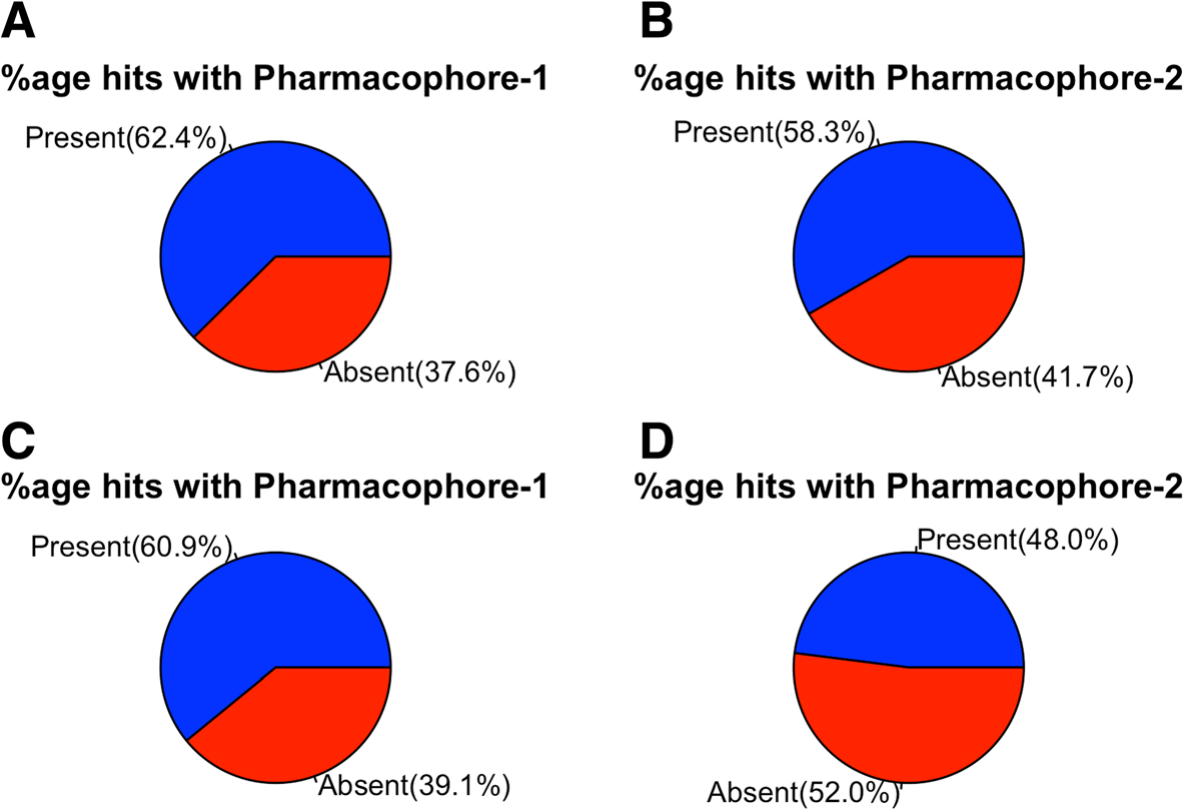
**Showing the results of pharmacophore based screening of both the datasets. A**) Represents Pharmacophore-1 properties in inhibitors of Rep_dataset; **B**) Showing the Pharmacophore-2 properties in inhibitors of Rep_dataset; **C**) Showing the Pharmacophore-1 properties in NRep_dataset inhibitors; **D**) Represents the Pharmacophore-2 properties in NRep_dataset inhibitors.

We have generated two pharmacophores using three first line and four second line *M.tb* drugs, (see Methods section) and then scanned these pharmacophores in our datasets. As shown in Figure 3, the screening of NRep_dataset inhibitors resulted in total 735 (60.9%) and 579 (48%) compounds for pharmacophore-1 and pharmacophore-2 respectively [Figure 3, Additional file 3: Table S2]. Similarly, screening of Rep_dataset for active compounds resulted in total 846 (62.4%), and 704 (58.3%) compounds for pharmacophore-1 and pharmacophore-2 respectively [Additional file 4: Table S3].

### Classification models

The PaDEL software used in this study calculates 881 PubChem, 166 MACCS, 79 EState, 307 SubStructure fingerprints and each corresponds to a specific substructure fragment. In this study, we have developed computational models on both the datasets using these fingerprints as described below.

### Model based on NRep_dataset

#### Model based on binary fingerprints

The first SVM based model that was developed using 881 PubChem fingerprints showed 65.09%, 62.33%, 63.89% sensitivity, specificity and accuracy with MCC value of 0.27 [Table 4]. Likewise, based on MACCS keys, we achieved best MCC value 0.15 using 43 fingerprints. As shown in Table 4, the performance of models developed using other fingerprints like MACCS, EState, and SubFP was poor with MCC values less than 0.2. Afterwards, we removed all fingerprints which have correlation > =0.8 to > =0.3 for all four classes and observed that the prediction accuracy is more or less similar up to correlation cutoff value 0.6 [Table 4, Additional file 5: Table S4]. In case of PubChem, our model showed 62.60%, 63.40%, 62.95% sensitivity, specificity and accuracy respectively with MCC value 0.26 at correlation cutoff 0.5. Similarly as shown in Additional file 5: Table S4, MACCS based 34 keys shows an accuracy value 55.93% with MCC value 0.12. In case of Estate based fingerprints, numbers of descriptors remained same from 0.6 to 0.4 cutoff, therefore no change in performance has been observed. In order to improve the performance, we developed a hybrid model using all reduced fingerprints at a correlation cutoff value > =0.6 obtained from each class and achieved MCC value 0.28 slightly better than model developed on individual class. However, using criteria of > =0.5, and > =0.4, the prediction accuracy decrease ~1% to 2% [Additional file 5: Table S4].

**Table 4:** Results of different binary fingerprints for NRep_dataset calculated from PaDEL software

**Fingerprint**	**Descriptor numbers**	**Sensitivity**	**Specificity**	**Accuracy**	**MCC**	**AUC**
**PubChem**	881	65.09	62.33	63.89	0.27	0.67
**PubChem (0.6)**	247	62.44	63.51	62.90	0.26	0.68
**MACCS**	166	56.63	59.20	57.75	0.16	0.60
**MACCS (0.6)**	36	53.07	58.99	55.64	0.12	0.57
**EState**	79	61.77	55.01	58.83	0.17	0.60
**EState (0.6)**	33	62.69	55.11	59.39	0.18	0.61
**SubFP**	307	59.12	60.60	59.77	0.20	0.63
**SubFP (0.6)**	96	57.63	61.79	59.44	0.19	0.63
**Hybrid (0.6)**	**412**	**65.67**	**62.00**	**64.07**	**0.28**	**0.69**

#### Model based on features selected using MCC and frequency based algorithms

A numbers of techniques are available for descriptors selection such as correlation based, genetic algorithm based, random forest based etc. In this study, we have used two feature selection techniques namely MCCA and frequency based for selecting highly informative fingerprints. In case of MCCA, the MCC value of each fingerprint was calculated and then used in arranging the fingerprints in term of increasing value of MCC, from this top 10, 15, and 20 fingerprints were selected. In case of frequency based algorithm, frequency of each fingerprint present in inhibitors and non-inhibitors was calculated as described in equation-1. Afterwards, top 10, 15, 20 features were selected on the basis of frequency difference in inhibitors vs. non-inhibitors (for each fingerprints) [Additional file 6: Table S5]. From these selected features, we observed that there is not much improvement in the performance of models with different number of features [data not shown]. The maximum MCC achieved on MCCA based method on top 15 is 0.18 while on top 15 frequency based method is 0.10 [Table 5]. Furthermore, development of hybrid model using selected descriptors from both methods resulted in slight increase in performance for each type of fingerprints. As shown in Table 5, a hybrid model developed using selected fingerprints by MCCA based method on all four classes shows accuracy 60.84% with MCC value 0.22 and AUC value 0.65.

**Table 5:** Results of different binary fingerprints for NRep_dataset on selected 15 descriptors calculated from PaDEL software

**Fingerprint**	**MCC-based descriptors**					**Frequency based descriptors**					**Hybrid (MCC + Frequency)**				
	**Sen.**^**a**^	**Spec.**^**b**^	**Acc.**^**#**^	**MCC**^**!**^	**AUC**^**!!**^	**Sen.**^**a**^	**Spec.**^**b**^	**Acc.**^**#**^	**MCC**^**!**^	**AUC**^**!!**^	**Sen.**^**a**^	**Spec.**^**b**^	**Acc.**^**#**^	**MCC**^**!**^	**AUC**^**!!**^
**PubChem**	59.37	58.45	58.97	0.18	0.67	60.03	41.01	51.76	0.01	0.51	**59.62**	**58.45**	**59.11**	**0.18**	**0.62**
**MACCS**	59.95	50.91	56.02	0.11	0.56	56.80	52.85	55.08	0.10	0.57	61.86	53.39	58.17	0.15	0.59
**EState**	56.97	55.76	56.44	0.13	0.59	55.80	54.47	55.22	0.10	0.58	59.54	53.07	56.72	0.13	0.59
**SubFP**	51.99	59.96	55.46	0.12	0.59	59.54	41.55	51.71	0.01	0.51	52.99	57.37	54.89	0.10	0.57
**Hybrid-4**	**59.45**	**62.65**	**60.84**	**0.22**	**0.65**	**61.28**	**57.05**	**59.44**	**0.18**	**0.60**	N.A	N.A	N.A	N.A	N.A

^a^*Sen*.: Sensitivity, ^b^*Spec*.:Specificity, ^#^*Acc*.:Accuracy, ^!^*MCC*: Matthews correlation coefficient, ^!!^*AUC*: Area Under Curve.

### Model based on Rep_dataset

#### Model based on binary fingerprints

**Figure 4:**
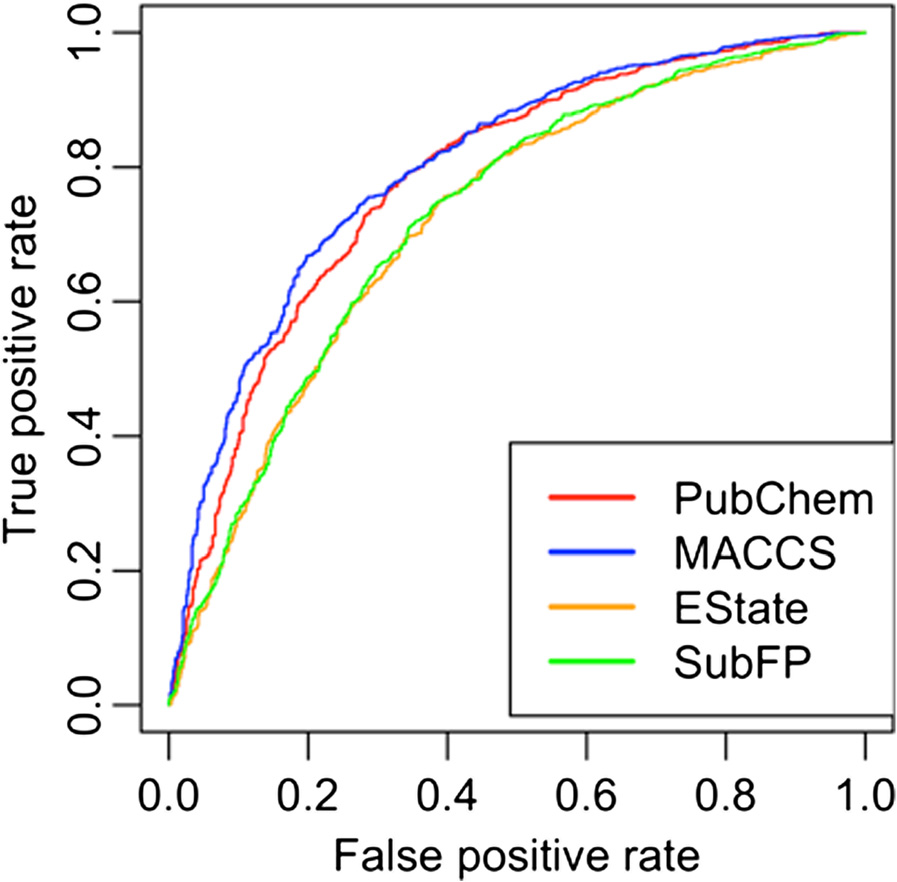
ROC plots of four class of fingerprints.

In this case, the 166 MACCSFP performed best with sensitivity/specificity 72.47%/73.97%, accuracy 73.02% with MCC value of 0.45 [Table 6]. The prediction accuracy of PubChem based fingerprint was nearly equal to MACCFP with MCC value of 0.44 [Figure 4]. However, the EState and SubFP was found to perform poor with MCC values of 0.34 and 0.35 respectively [Table 6, Figure 4]. As shown in Additional file 5: Table S4, using MACCS fingerprints at 0.6 cutoff, our model showed an accuracy value 73.02% while a decrease had been observed at correlation cutoff value 0.5. However, in case of Estate, and SupFP, the numbers of descriptors were more or less constant, therefore no significant increment or decrement in performance was observed. From these results, we concluded that reduction of fingerprints at correlation cutoff value 0.6 is sufficient for attribute selection [Table 6, Additional file 5: Table S4]. As shown in Table 6, the hybrid model has increased the sensitivity ~3% to 4% but the prediction accuracy was nearly same as that of MACCS fingerprints based classification model.

**Table 6:** Results of different binary fingerprints for Rep_dataset calculated from PaDEL software

**Fingerprint**	**Descriptor numbers**	**Sensitivity**	**Specificity**	**Accuracy**	**MCC**	**AUC**
**PubChem**	881	75.79	68.97	73.30	0.44	0.78
**PubChem (0.6)**	247	73.06	72.18	72.74	0.44	0.80
**MACCS**	166	72.47	73.97	73.02	0.45	0.80
**MACCS (0.6)**	91	73.36	72.44	73.02	0.44	0.79
**EState**	79	70.92	63.59	68.24	0.34	0.72
**EState (0.6)**	33	70.77	64.10	68.34	0.34	0.72
**SubFP**	307	70.63	65.64	68.81	0.35	0.73
**SubFP (0.6)**	96	66.49	68.33	67.17	0.34	0.72
**Hybrid-4 (0.6)**	467	75.72	68.87	73.58	0.45	0.78

#### Model based on features selected using MCC and frequency based algorithms

The above described technique was also applied for selection of descriptors. As shown in Table 7, the classification model on MCC based selected fingerprints shows sensitivity ranges 53% to 67%, specificity 56% to 64% and MCC value 0.16 to 0.28. The hybrid model of MCC dependent fingerprints encapsulated the features of all four classes show significant improvement in MCC value from 0.28 to 0.35 [Table 7]. However, the frequency-based model performed poor in this dataset as well [Additional file 7: Table S6]. In frequency based selected fingerprints, estate fingerprints shows sensitivity of 64.13%, specificity of 58.59%, accuracy of 62.11% with AUC value of 0.64. The four-hybrid model (for each class) developed using selected fingerprints from both the MCC and frequency based methods also resulted in slight improvement in performance.

**Table 7:** Results of different binary fingerprints for Rep_dataset on selected 15 descriptors calculated from PaDEL software

**Fingerprint**	**MCC-based descriptors**					**Frequency based descriptors**					**Hybrid (MCC + Frequency)**				
	**Sen.**^**a**^	**Spec.**^**b**^	**Acc.**^**#**^	**MCC**^**!**^	**AUC**^!!^	**Sen.**^**a**^	**Spec.**^**b**^	**Acc.**^**#**^	**MCC**^**!**^	**AUC**^**!!**^	**Sen.**^**a**^	**Spec.**^**b**^	**Acc.**^#^	**MCC**^**!**^	**AUC**^**!!**^
**PubChem**	59.85	56.92	58.78	0.16	0.61	60.00	41.15	53.11	0.01	0.51	58.60	57.44	58.17	0.15	0.61
**MACCS**	53.95	58.85	55.74	0.12	0.58	54.39	53.72	54.15	0.08	0.54	64.35	61.15	63.19	0.25	0.66
**EState**	67.31	59.74	64.54	0.26	0.66	64.13	58.59	62.11	0.22	0.64	66.86	58.85	63.93	0.25	0.65
**SubFP**	64.43	64.10	64.31	0.28	0.65	59.70	44.62	54.19	0.04	0.53	**66.20**	**63.33**	**65.15**	**0.29**	**0.66**
**Hybrid-4**	**72.55**	**62.82**	**68.99**	**0.35**	**0.73**	**66.13**	**59.10**	**63.56**	**0.25**	**0.67**	N.A	N.A	N.A	N.A	N.A

^a^*Sen*.: Sensitivity, ^b^*Spec*.:Specificity, ^#^*Acc*.:Accuracy, ^!^*MCC*: Matthews correlation coefficient, ^!!^*AUC*: Area Under Curve.

## Discussion

In contrast to the general antibacterial rules or models, there is no report for phase specific rules and very limited efforts have been made to derive such ‘rules’ for tuberculosis [32-35]. Therefore, in the present study, we tried to generate new phase specific rules for better inhibitor predictions and drug development against *M.tb.* Our analysis suggested that simple molecular properties of chemical compounds like molecular weight, logP, polar surface area etc. were playing an important role in crossing the mycobacterium cell wall and its killing. Based on this study, we propose that some properties like molecular weight of compounds >300 Da for replication inhibitors and <380 Da for compounds inhibiting tuberculosis growth in non-replication mode. Based on this study, we derived some rules for identifying inhibitors against *M.tb* (for details see Results section). We have also shown that some substructure patterns like nitro, alkyne, enamine were dominating in inhibitor class of both phases. Similarly, the substructure like amine, tertiary_carbon, alkylarylthioether, secondary_carbon were not preferred in any of the growth phase inhibitors. This study demonstrated that molecules targeting the replicative and non-replicative phases have different chemical and molecular properties. These variations could arise from differences in the cellular metabolism and composition of cell wall of *M.tb* in these two phases of pathogenic cycles. We also observed that out of 7 drugs on an average 3 satisfied these criteria for replication inhibitors and out of 4 drugs known to be active in latent phase, ~2 also satisfied these rules implying the applicability of these modified rules for identifying anti-tuberculosis molecules. However this observation also suggests that there is an urgent requirement to increase the dataset of antitubercular drugs to further improve these rules. As suggested previously, identification of the undesirable fragment is important in early stages of drug discovery to reduce the time and cost involved in optimization process [36]. Our SMART filtering results are similar to that of previous studies. The substructure patterns, identified in this work will be helpful for TB research community to design most potent inhibitory molecules against *M.tb.*

Additionally, four types of binary fingerprints were used to develop classification models using SVM based machine learning approach. We observed that the reduction of descriptors even at > =0.6 correlation cutoff, is sufficient to develop a robust classification model. As reported in different studies, we also observed that descriptors selection was playing an important role in efficient model building [17,18]. In the present work, we have introduced a new algorithm named MCCA (Matthews Correlation Coefficient Algorithm) for selection of informative descriptors/fingerprints.

In past, different studies have been done to predict *M.tb* inhibitors. The Bayesian based classification model developed by Ekins *et. al.* has good predictive power value >0.7 (in-term of AUC) on independent dataset [9]. In 2011, another Bayesian based model was developed to differentiate inhibitors under aerobic vs anaerobic condition [8]. But the major limitation of previous studies was that these were not able to predict the replication/non-replication phase specific inhibitors of *M.tb* based on carbon starvation model. In 2010, A report by Gengenbacher *et. al* showed that the behaviour of drugs like steptomycin, rifampicin, isoniazid etc. was entirely different in replication, hypoxia induced drug tolerant and nutrient depleted models [37]. Secondly, these models were based on the use of commercial softwares, hence limiting their accessibility. Similarly, in 2011, Periwal *et. al.* developed model on three dataset with maximum MCC value of 0.52. In 2012, a computational model was developed using large datasets obtained from high throughput screening based on whole cell screening using microdilution alamar blue assay and achieved maximum AUC value of 0.748 [38]. Although, Periwal *et. al.* used the free softwares for model development but the non-availability of free software/webserver of these study restrict the use of their model by the scientific community. Considering these observations, we have developed a computational model that could discriminate the active compounds from inactive ones in both phases. Based on this study, we have also developed a user friendly, freely available webserver to search for new active molecules. We anticipate that these findings will provide insight that could be used in future to identify novel inhibitors effective against *M.tb* in either replicative or non-replicative phase.

In summary, we have identified some important substructures that are present in *M.tb* inhibitors. The SMART based filtering had identified 164 compounds from replicative inhibitors dataset and 180 compounds from non-replicative inhibitors dataset that passed all these three filters (see Results section) would be useful in future to reduce the effect of poor ADMET properties. These compounds would be useful in future for virtual screening and designing new inhibitors against *M.tb.* This study is implemented in the form of open source webserver to assist scientific researcher, and to boost up the drug discovery process against *M.tb.*

### Web service to community

One of the main reasons of slow progress in Computer Aided Drug Designing (CADD) is the lack of freely available softwares and its implementation in user-friendly webservers. Most of these studies were focused on commercial softwares and hence their implementation is difficult. Our major emphasis is to help scientific community by developing freely accessible webserver/softwares based on our study. Thus, we have used both commercial as well as open source softwares in this study. Based on that, we have developed a webserver using SVM based classification model. Additionally, we have implemented the pharmagist software for identifying pharmacophore features similar to the first line and second line anti-mycobacterial drugs. Server has been developed under Linux environment using CGI-Perl scripts. In this web server, there are three options for molecule submission, 1) Draw structure using JME editor (http://www.molinspiration.com/jme/), 2) By pasting molecule in mol/mol2 file format, 3) By file upload. The results of prediction is provided in the tabular format with prediction class (inhibitor or non-inhibitor) of both phase as well as pharmacophore features similar to first line as well as second line *M.tb* drugs present or absent.

## Abbreviations

QSAR: Quantitative structural activity relationship; SMARTS: SMiles ARbitrary target specification; MLSMR: Molecular libraries small molecule repository; AUC: Area under curve; SVM: Support vector machine.

## Competing interests

The authors declare that they have no competing interests.

## Authors’ contribution

DS carried out the data analysis and interpretation, developed computer programs, webserver and wrote the manuscript. AK helped in data processing and analysis. GPSR, RT conceived and coordinated the project, guided its conception and design, helped in the interpretation of data, refined the drafted manuscript and gave overall supervision to the project. All authors have read and approved the final manuscript.

## Supplementary Material

Additional file 1: Figure S1–S8 Physicochemical properties distribution of active and decoys molecules. Click here for file

Additional file 2: Table S1 Simple molecular properties of *M.tb* inhibitors and their rules based classification. Click here for file

Additional file 3: Table S2 Pharmacophore based screening score of NRep_dataset. Click here for file

Additional file 4: Table S3 Pharmacophore based screening score of Rep_dataset. Click here for file

Additional file 5: Table S4 Prediction results at different cutoff value for both Rep_dataset and NRep_dataset. Click here for file

Additional file 6: Table S5 Frequency based distribution of different classes of fingerprints between active and inactive compounds of NRep_dataset. Click here for file

Additional file 7: Table S6 Frequency based distribution of different classes of fingerprints between active and inactive compounds of Rep_dataset. Click here for file
