# Supplementary material for: Designing of inhibitors against drug tolerant Mycobacterium tuberculosis (H37Rv)
Source: Chem Cent J. 2013 Mar 8;7:49. doi: 10.1186/1752-153X-7-49 (PMC3639817; doi:10.1186/1752-153X-7-49)
Supplement: Additional file 1: Figure S1–S8 — Physicochemical properties distribution of active and decoys molecules. [file 1752-153X-7-49-S1.pdf]

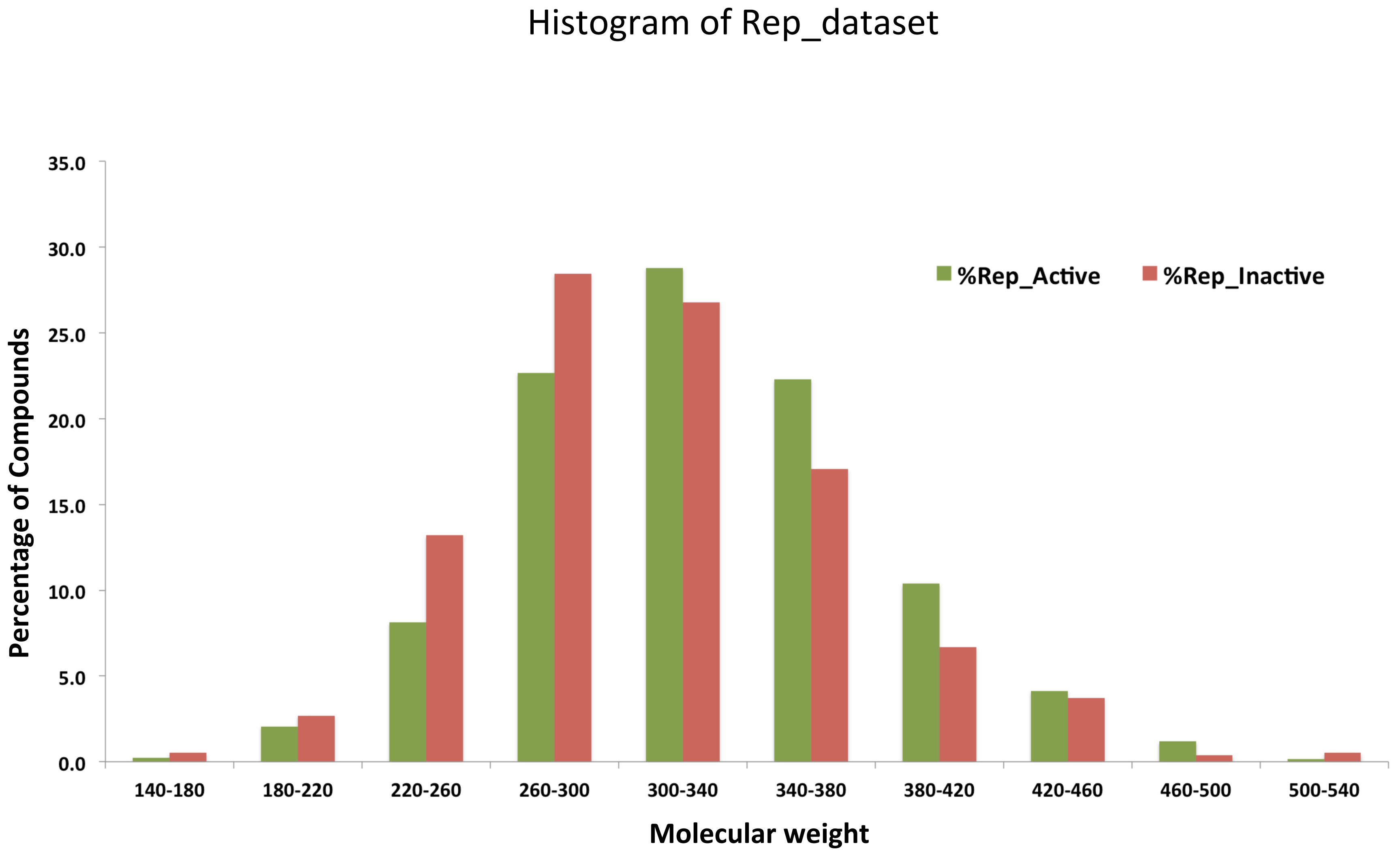
Histogram of Rep_dataset
35.0
30.0
25.0
20.0
15.0
10.0
5.0
0.0
Percentage of Compounds
%Rep_Active
%Rep_Inactive
140-180
180-220
220-260
260-300
300-340
340-380
380-420
420-460
460-500
500-540
Molecular weight

Figure-S1

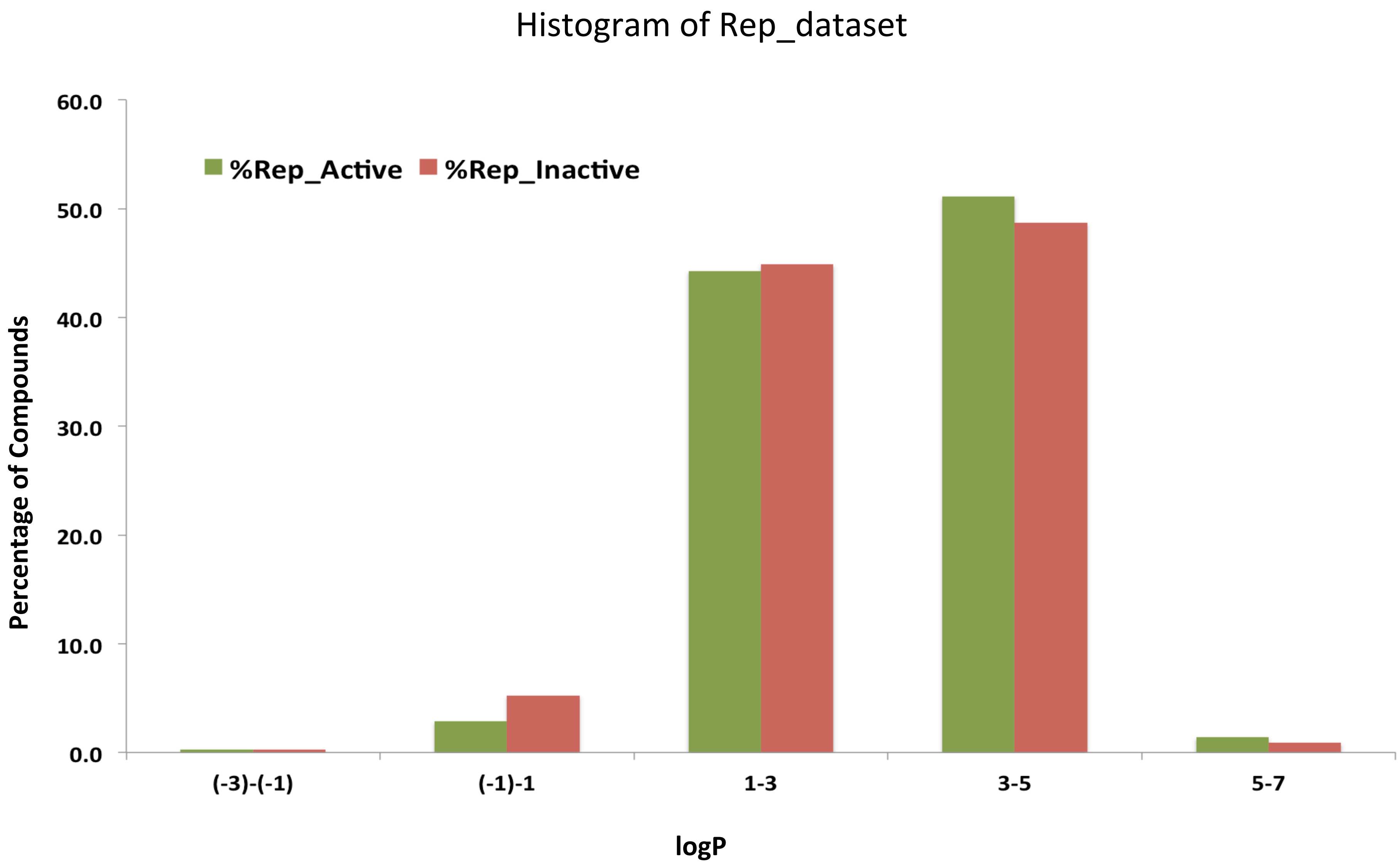
Histogram of Rep_dataset
%Rep_Active
%Rep_Inactive
60.0
50.0
40.0
30.0
20.0
10.0
0.0
Percentage of Compounds
(-3)-(-1)
(-1)-1
1-3
3-5
5-7
logP

Figure-S2

Histogram of Rep_dataset

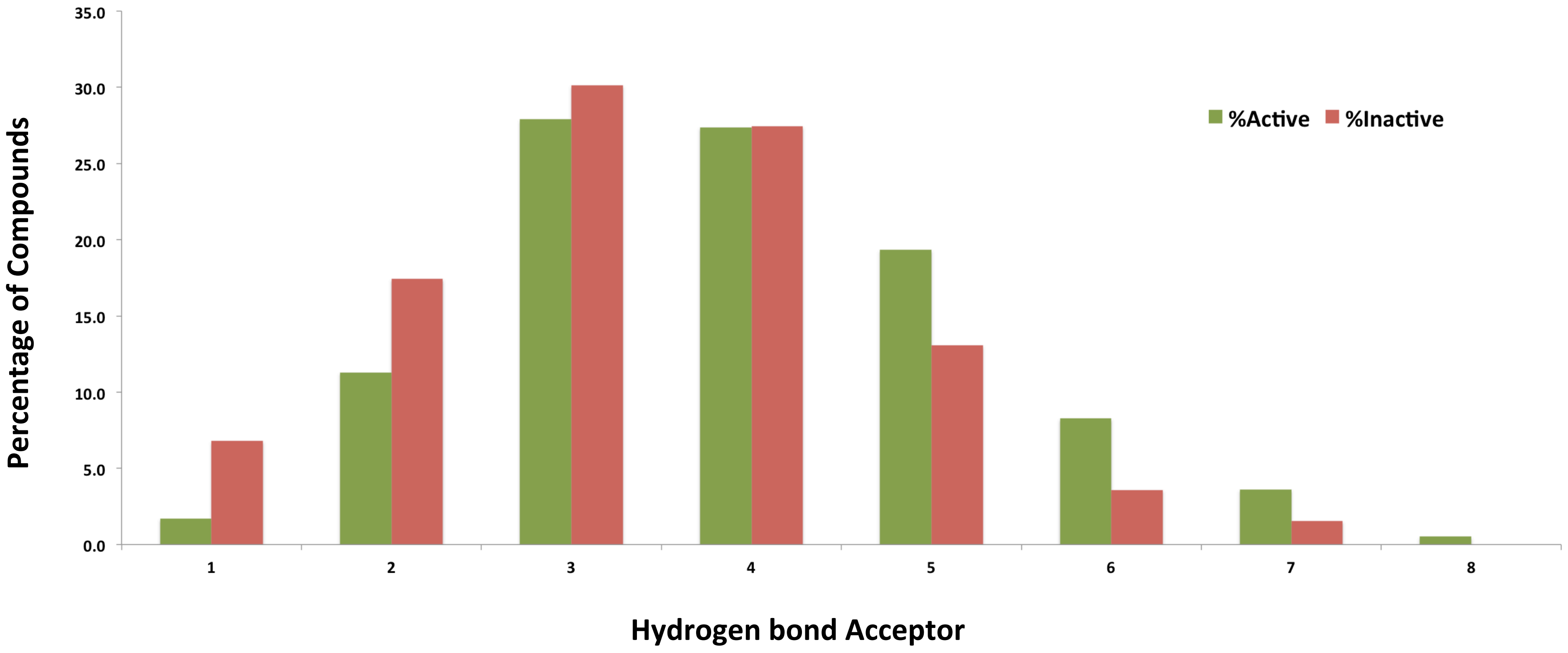

Figure-S3

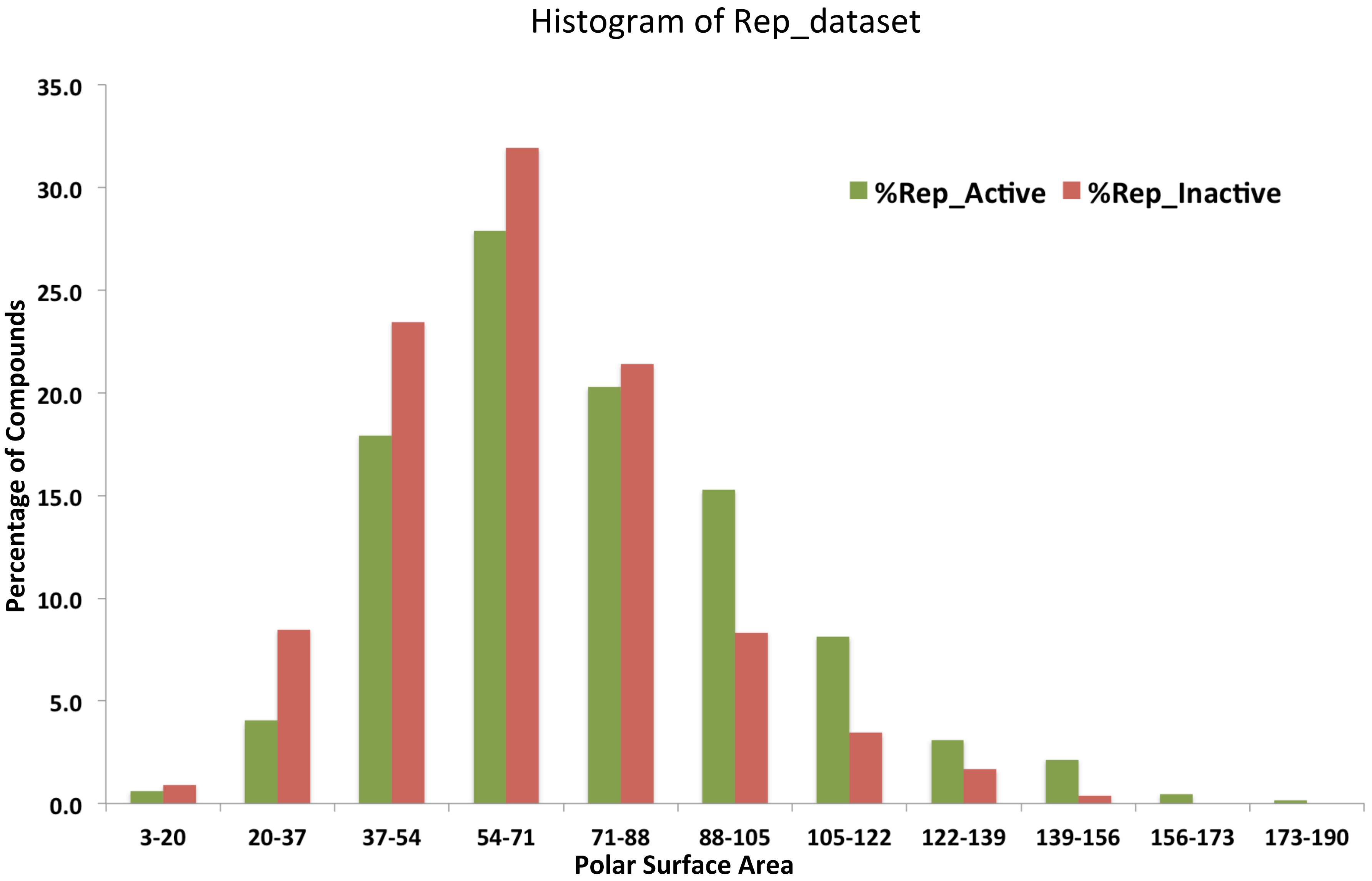
Histogram of Rep_dataset
%Rep_Active
%Rep_Inactive
Percentage of Compounds
35.0
30.0
25.0
20.0
15.0
10.0
5.0
0.0
3-20
20-37
37-54
54-71
71-88
88-105
105-122
122-139
139-156
156-173
173-190
Polar Surface Area

Figure-S4

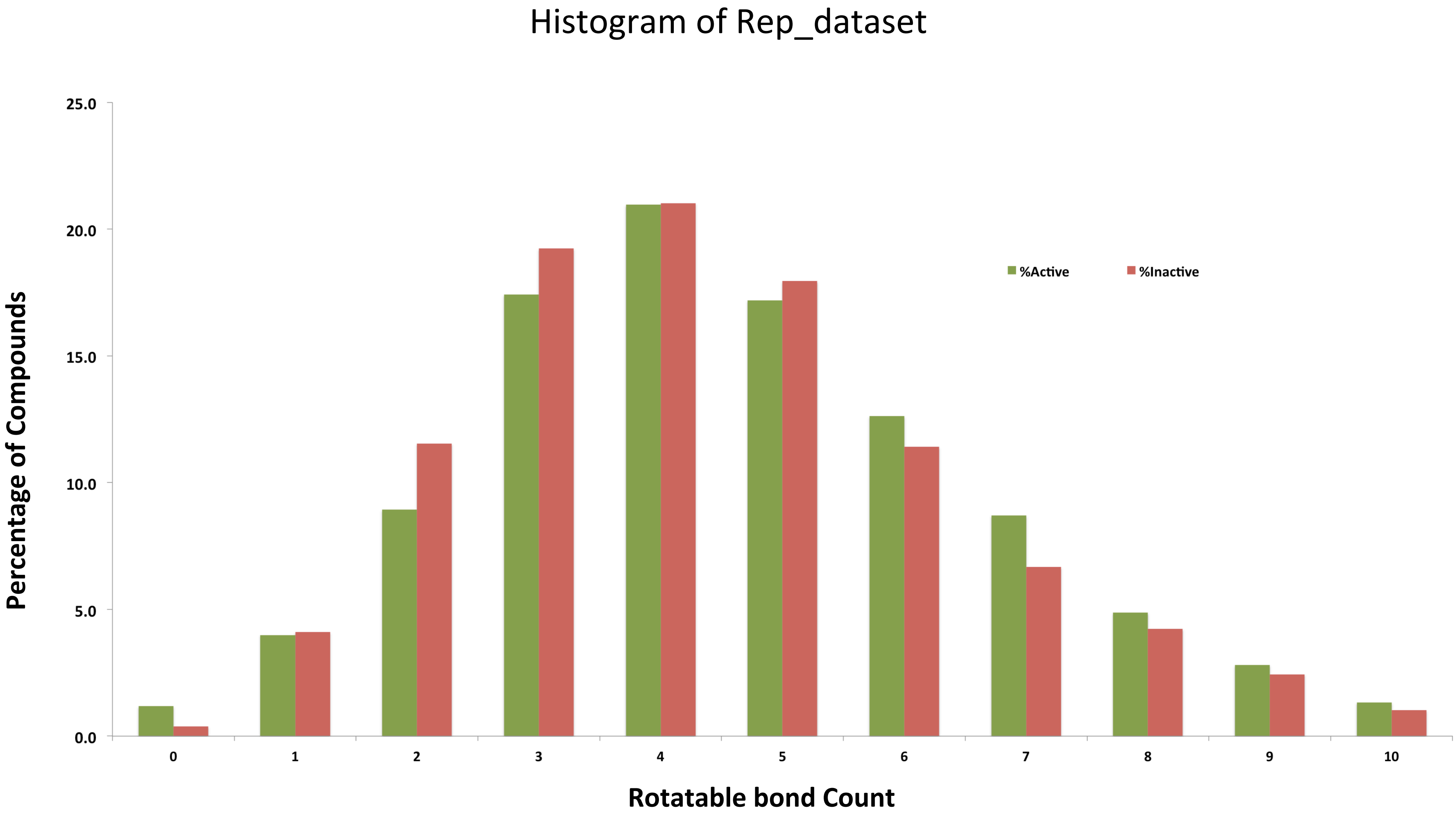
Histogram of Rep_dataset
25.0
20.0
15.0
10.0
5.0
0.0
Percentage of Compounds
%Active
%Inactive
0
1
2
3
4
5
6
7
8
9
10
Rotatable bond Count

Figure-S5

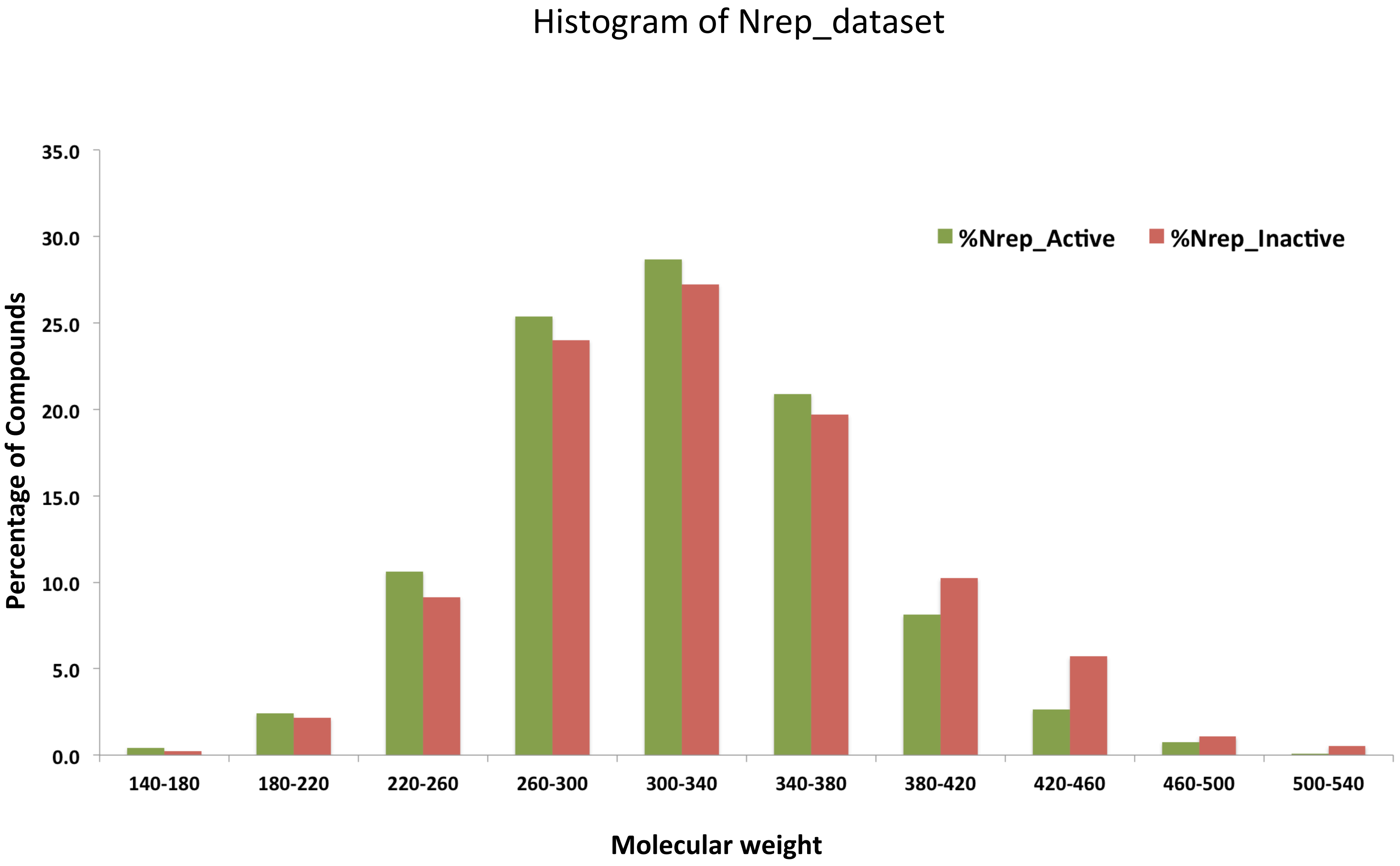
Histogram of Nrep_dataset
%Nrep_Active
%Nrep_Inactive
Percentage of Compounds
35.0
30.0
25.0
20.0
15.0
10.0
5.0
0.0
140-180
180-220
220-260
260-300
300-340
340-380
380-420
420-460
460-500
500-540
Molecular weight

Figure-S6

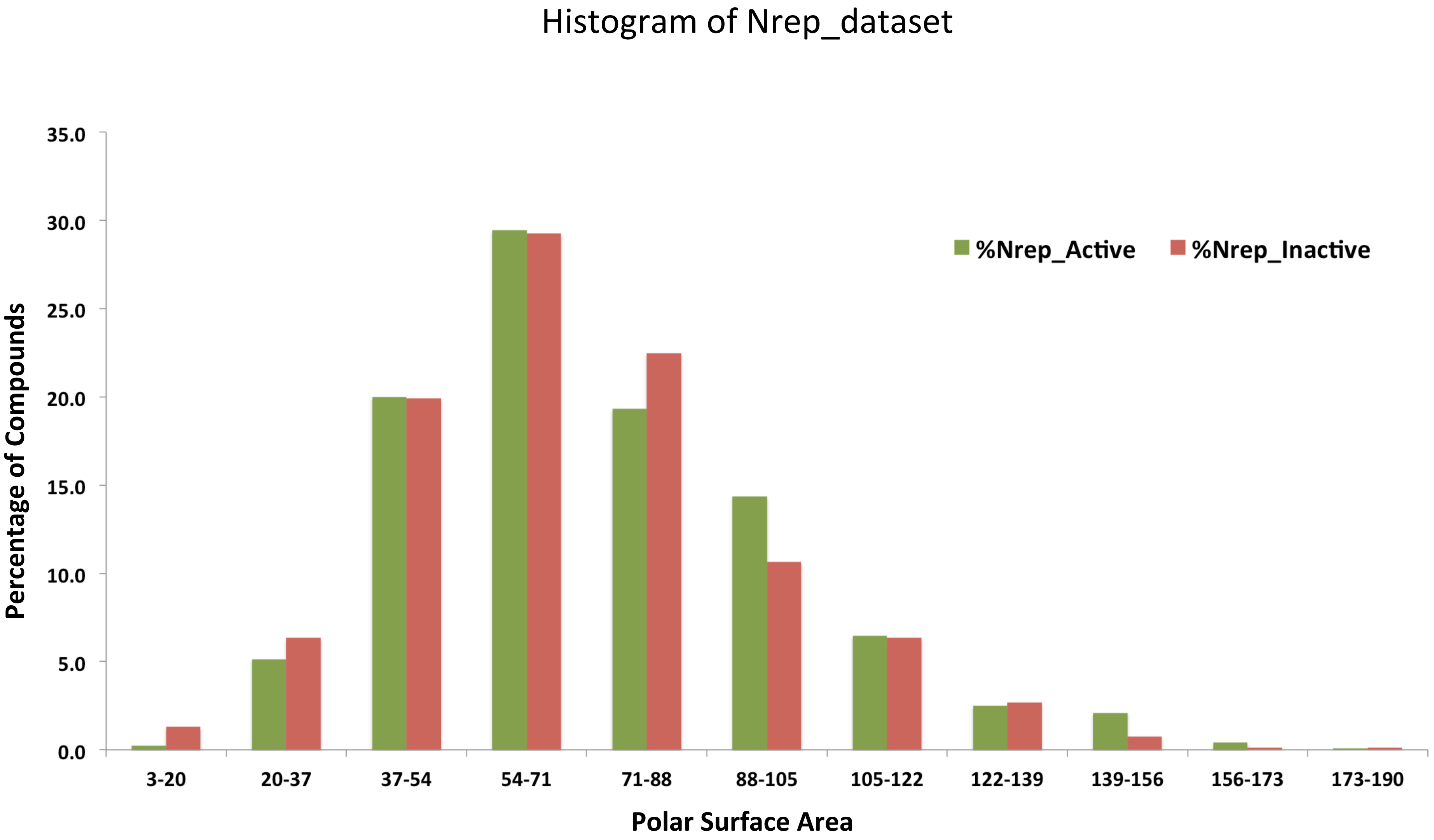

Histogram of Nrep_dataset
35.0
30.0
25.0
20.0
15.0
10.0
5.0
0.0
Percentage of Compounds
%Nrep_Active
%Nrep_Inactive
3-20
20-37
37-54
54-71
71-88
88-105
105-122
122-139
139-156
156-173
173-190
Polar Surface Area

Figure-S7

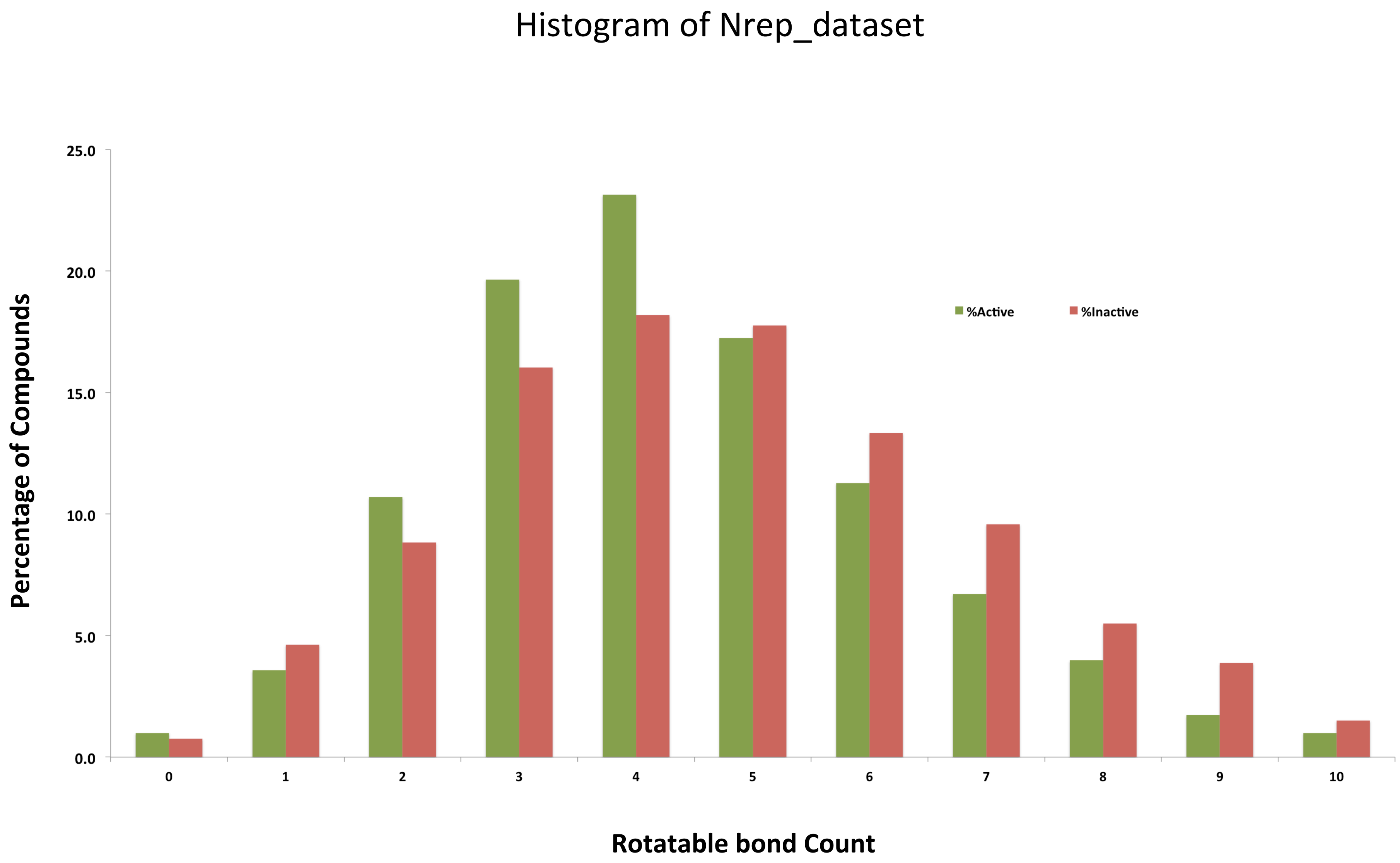
Histogram of Nrep_dataset
25.0
20.0
15.0
10.0
5.0
0.0
Percentage of Compounds
%Active
%Inactive
0
1
2
3
4
5
6
7
8
9
10
Rotatable bond Count

Figure-S8
